# Evaluation of Skin Prick-Test Reactions for Allergic Sensitization in Dogs With Clinical Symptoms Compatible With Atopic Dermatitis. A Pilot Study

**DOI:** 10.3389/fvets.2019.00448

**Published:** 2019-12-17

**Authors:** Ana M. Carmona-Gil, Jorge Sánchez, Juan Maldonado-Estrada

**Affiliations:** ^1^One Health and Veterinary Innovative Research and Development Group, Faculty of Agrarian Sciences, School of Veterinary Medicine, University of Antioquia, Medellin, Colombia; ^2^Centro de Dermatología Veterinaria DermaVet, Medellin, Colombia; ^3^Grupo de Alergología Clínica y Experimental, Facultad de Medicina, IPS Universitaria, Universidad de Antioquia, Medellin, Colombia

**Keywords:** allergens, atopic, canine, dermatitis, intradermal reaction, prick test

## Abstract

Skin prick-test is the first choice for the detection of type I hypersensitivity in human atopic dermatitis. Canine atopic dermatitis resembles several symptoms of the disease in the human counterpart. In canine atopic dermatitis, intradermal testing is the test of choice, and there are few reports on the use of skin prick test (SPT) in dogs. The purpose of this study was to evaluate SPT reactions in atopic dogs and a healthy control group to 11 environmental allergens. Eleven glycerinated allergens were applied on the left lateral thorax of nine atopic dogs and nine healthy dogs. The skin was pricked with a feather lancet and evaluated for the positive percutaneous reaction at 5, 10, 15, and 20 min after the application of the allergens. Data were analyzed by the Shapiro-Wilk test to test for normal distribution. Data that did not meet normality were analyzed by a one-sided Wilcoxon ranked sum test with a *p*-value of 0.05. Six out of 9 atopic dogs tested positive for at least one of the allergens tested. None of the dogs in the control group showed a positive reaction to the allergens included in the test. *Blomia tropicalis, Dermatophagoides farine*, and *Dermatophagoides pteronyssinus* exhibited the highest reaction rate among the group of atopic dogs. There was not a statistical difference in the histamine reaction (positive control) between both groups. In this set of dogs, the test exhibited a 100% specificity and a sensitivity of 66%. The use of skin prick-test in the detection of causative allergens of human atopic dermatitis has proved to be a sensitive and specific tool frequently used by human allergists. Due to the number of similarities in canine and human atopic dermatitis, this could be a valuable tool that needs intensive research in veterinary medicine. The published research so far correlates to the results obtained in this investigation. However, future studies evaluating the concordance between *in vitro* specific IgE antibody assays and SPT must be carried out simultaneously to validate the test.

## Introduction

The skin prick test (SPT) protocol is a method used in human medicine for the diagnosis of IgE-mediated allergic diseases (1). The result of SPT is a type I hypersensitivity reaction in the skin caused by allergens of environmental or food origin. The presence and degree of cutaneous reactivity provide the interpretation grounds for SPT. When the patient's skin is exposed to an allergen he/she has been previously sensitized to, the binding of allergen to its specific IgE anchored to IgE receptor (IgE-R) on the cell surface of mast cells triggers their immediate degranulation and histamine release (2). This reaction will release innate immunity inflammatory mediators that produce a wheal and flare response that can be observed and quantitated directly (3–5). The clinical history and previous allergen exposure of the patient are relevant for the selection of the allergens to be evaluated in the test (6, 7). The reaction to each allergen is restricted to the immediate area of the SPT, allowing many different allergens to be tested at the same time. Results are retrieved within 15 min after taking the exam, providing immediate interpretation (8). In humans, SPT is approved as the primary diagnostic test for IgE-mediated allergic diseases in Europe, the United States, and South America. The other diagnostic tests are the quantification of allergen-specific IgE in serum and the intradermal test (IDT). The advantages that SPT has over these two other tests are the low costs, rapid interpretation of results, safety, and higher specificity (9), and it appears to be significantly less painful.

SPT as a screening method is frequently used in humans for the diagnosis of allergens causing atopic dermatitis (AD) (9). Dogs also suffer from AD, which is one of the most prevalent skin diseases in this species, with up to 10% of the general population being affected (10). Even though it has been suggested that dogs are a suitable model for studying human AD (11), the canine AD is characterized as a genetically predisposed chronic and pruritic skin disease. Alike the human counterpart, canine AD presents a Th2 skewed response in the acute phase and a mixed Th1/Th2/Th17/Th22 response in the chronic stage of the disease (12). It is also predominantly IgE-mediated and has similar lesional distribution patterns, mainly on the face, paws, and flexural folds (13). The test of choice in the diagnosis of canine AD is the specific serum IgE or the IDT (14, 15). The earliest report in the literature on the use of SPT in dogs dates to 1991 (16), where authors found that IDT test reactions were identifiable and that the SPT tests did not give easily interpretable responses. Currently, only the work by Ballauf (16) reported the use of SPT in dogs suffering from dermal or respiratory problems, whereas two other studies reported the results of SPT in non-allergic dogs (17, 18). More studies are warranted to evaluate the usefulness of SPT in the causative allergen detection of AD together with the correlation of the serum levels of specific IgE to each allergen tested (19). The objective of this study was to explore the SPT in dogs with atopic dermatitis and compared the results with a control group providing evidence on their usefulness for the diagnosis of allergen etiology in dos suffering AD.

## Materials and Methods

### Dogs

Nine client-owned dogs with a clinical diagnosis of canine AD (experimental group) and nine non-atopic dogs were used as healthy (control group) dogs (Table 1). All dog owners agreed to place their dogs in the study and gave full informed consent. The atopic dogs had to fulfill the following inclusion criteria: (i) Patients with a chronic history of pruritic skin disease that meet at least 5 of Favrot's criteria (i.e., age at onset <3 years, mostly indoor, corticosteroid-responsive pruritus, chronic or recurrent yeast infections, affected front feet, affected ear pinnae, non-affected ear margins, non-affected Dorso-lumbar area, and “non-lesional” pruritus at onset) (20); (ii) Onset of disease from 1 to 5 years of age; (iii) No food allergies through a strict 6 week food trial with 3 week boost; (iv) Up to date flea and tick prevention; (v) No secondary staphylococcal or yeast infections through skin cytology; and (vi) Have no oral, topical or injectable glucocorticoids, cyclosporine or oclacitinib for a minimum of 3 weeks prior to the study. Control dogs were: (i) 1–10 years of age; (ii) had no history of allergic diseases; (iii) had no topical, oral, or injectable requirements 3 weeks before the test; (iv) had typical results at the current physical exam; and (v) had no remarkable previous medical history.

**Table 1:** Breed and age of dogs with AD and dogs of the control group included in the study.

**AD group**		**Control group**	
**Breed**	**Age (years)**	**Breed**	**Age (years)**
English bulldog	1	Mixed breed	5
French bulldog	3	Mixed breed	8
Beagle	9	Mixed breed	8
West highland white terrier	3	Mixed breed	9
Springer spaniel	1	Afghan	2
Maltese	4	Pitbull	3
Cocker spaniel	2	Standard schnauzer	7
Labrador retriever	7	Standard schnauzer	4
Yorkshire terrier	8	Bull terrier	2
Average (± Standard error)	4.2 ± 1.0		5.3 ± 0.9

### Skin Prick Test

**Figure 1:**
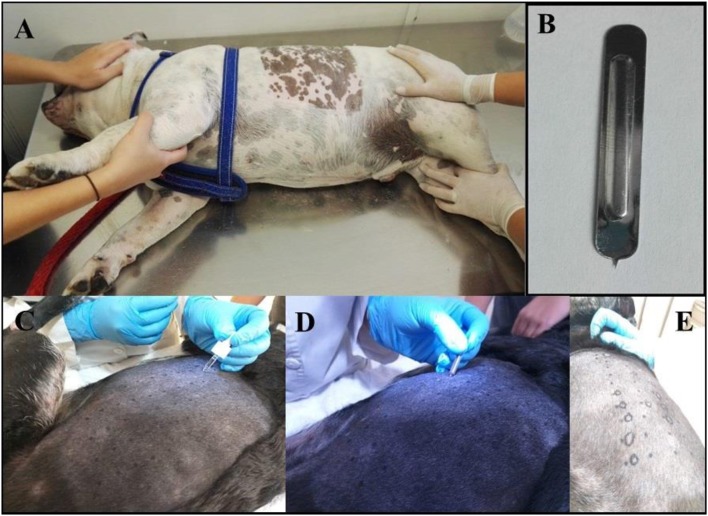
**(A)** Photograph showing the patient being held down manually. It is shown the area of the lateral thorax clipped, where the prick test was performed. **(B)** The Spricker used in the study showing the 1 mm prick. **(C)** A drop of each allergen, positive and negative control, were placed in the prepared aseptic skin in marker dots. **(D)** Pricking of the skin in the allergen-containing drops. **(E)** The resulting wheal shape was delineated with a marker for its measurements and interpretation.

**Figure 2:**
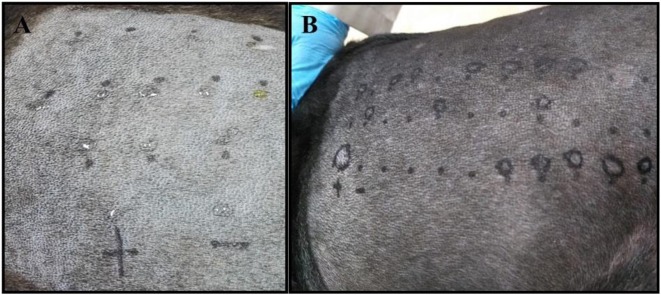
**(A)** The skin of a negative control patient with the drops of each allergen applied before pricking the skin is shown. **(B)** The skin of a positive dog exhibiting several degrees of reaction to different allergens, including the positive control reaction (+ mark).

All of the patients were required to be bathed with a 2% Chlorhexidine solution at the most 2 days before the test. The lateral thorax was clipped with a 40 blade (Figure 1A). The skin surface was then cleaned with 70% isopropyl alcohol before the application of the allergens (Figure 1D). The test sites were marked using a permanent marker with a 2 cm separation between sites and a 5 cm separation between the histamine and control solution (Figures 2A,B). A kit used for the diagnosis of human AD, containing 11 allergens (Table 2), was used (ALK Allergologisk Laboratorium A/S, Hørsholm, Denmark). Allergens were placed on each designated site (Figure 1C), a feather metal lancet having angular shoulders and a small 1 mm pricker (Figure 1B) was used for inoculating each allergen and discarded afterward, and the skin was pricked at a 45° angle (Figure 1D). A 10 mg/ml di-hydrochloride glycerinated histamine base was applied as a positive control, and a 50% glycerol saline solution was used as the negative control. After pricking the skin, the allergen-containing drops were removed simultaneously with a paper towel. Wheals were evaluated at 5, 10, 15, and 20 min after the test. Each wheal was assessed for the presence of erythema (Figure 1E), and the average orthogonal wheal was measured with a metric ruler. The allergens tested with their respective concentrations are listed in Table 2. A reaction was considered positive when the diameter of the wheal was equivalent to or >3 mm, which is the minimum average among the width of the wheals of the positive and negative controls. None of the dogs required sedation during the procedure and were held down manually. Highlighting and palpation were performed for a better delimitation of the reaction zone when necessary.

**Table 2:** Allergens^*^ used in the study and their corresponding concentration.

**Allergen**	**Description**	**Concentration**
Alternaria	*Alternaria* glycerinated extract	3 μg/ml
Aspergillus	*Aspergillus fumigatus* glycerinated extract	25 μg/ml
Artemisia	*Artemisia vulgaris* glycerinated extract	50 mg/ml
Cupressus arizona		10 mg/ml
Grass mix	(*Poa pratensis, Dactylis glomerata, Lolium perenne, Phleum pratense, Festuca pratensis, Helictotrichon pretense*) glycerinated extracts	50 mg/ml
*Cynodon dactylon*	*Cynodon dactylon* glycerinated extract	50 mg/ml
*Dermatophagoides farinae*	*Dermatophagoides farina* glycerinated extract	100 mg/ml
*Dermatophagoides pteronyssinus*	*Dermatophagoides pteronyssinus* glycerinated extract	100 mg/ml
*Blomia tropicalis*	*Blomia tropicalis* glycerinated extract	150 mg/ml
Fire ant	Fire ant glycerinated extract	1:100 w/v
Cat epithelium	*Felis domesticus* skin glycerinated extract	10.000 BAU/ml
Histamine (Positive Control)		10 mg/ml
Diluted glycerol-saline solution (Negative Control)		1:20 w/v

* *Source: (ALK Allergologisk Laboratorium A/S, Hørsholm, Denmark)*.

### Statistical Analysis

The sensitivity and specificity of the test were calculated under the following formulas: Sensitivity = true positive/(true positive + false negative) × 100. Specificity = True negative/(True negative + False positive) × 100. Histamine reactions were compared between the AD and control groups. The Shapiro-Wilk test was performed for the evaluation of normality. Score data were ordinal and not normally distributed. The one-sided Wilcoxon ranked sum test was used with a *p*-value of 0.05. The *U*-value (stands for unbiased) determines whether the observed U, in this case, supports the null or research hypothesis. This is done by determining a critical value of *U* such that if the observed value of *U* is less than or equal to the critical value, we reject H0 in favor of H1 and if the observed value of *U* exceeds the critical value we do not reject H0.

## Results

### Dogs With Atopic Dermatitis

**Figure 3:**
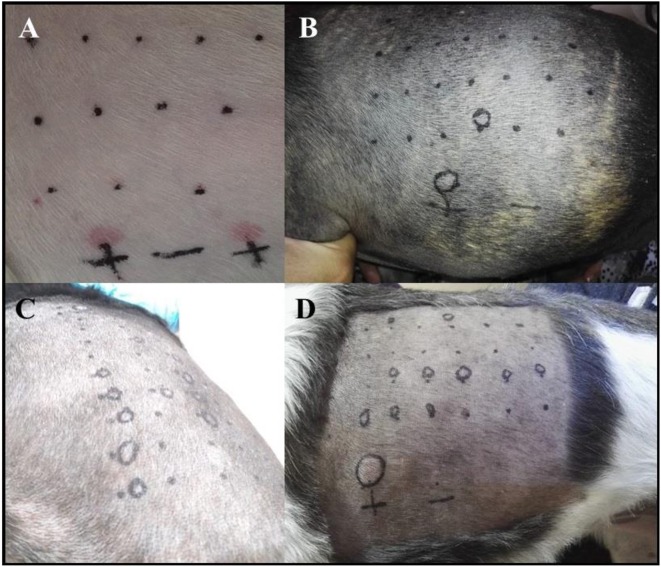
**(A)** Photograph showing the PT in one of the AD patients with a negative reaction to all of the allergens tested and with a double positive reaction to the histamine control solution. **(B–D)** The skin of a positive dog to a single **(B)** or several **(C,D)** allergens.

Six out of nine dogs (66.6%) with a clinical diagnosis of the canine AD tested positive to at least one of the allergens tested (Figures 3B–D). The remainder three dogs did not react to any of the allergens tested, but had a positive reaction to the histamine control, validating the test. None of the AD dogs responded against Alternaria, Aspergillus, Artemisia vulgaris, or Cat epithelium. The allergens Cupressus Arizona, Grass mix, Cynodon dactylon, and Fire ant had two dogs reacting positively for each allergen. *Dermatophagoides farinae* and *Dermatophagoides pteronyssinus* had four dogs, responding positively to each allergen. *Blomia tropicalis* had five dogs with a positive reaction (Table 3).

**Table 3:** The diameter of wheal in AD dogs that reacted positively to specific allergens by SPT.

**Breed of the dog**	**Allergen source and wheal reaction (mm)**						
	***D. farinae*** ** (mm)**	***D. pteronyssinus*** ** (mm)**	***Blomia tropicalis*** ** (mm)**	**Fire ant** ** (mm)**	***Cynodon*** ***dactylon* (mm)**	**Cupressus** ** Arizona (mm)**	**Grass mix** ** (mm)**
French bulldog	–	–	3	–	–	–	–
English bulldog	8	7	5	3	–	–	–
Beagle	5	3	3	–	3	3	4
West Highland White Terrier	–	–	–	6	–	–	–
Cocker spaniel	6	5	5	–	–	–	–
Yorkshire terrier	4	3	3	–	3	3	3
Wheal diameter (average ± standard error)	5.75 ± 0.9	4.5 ± 1.0	3.8 ± 0.5	4.5 ± 1.5	3 ± 0	3 ± 0	3.5 ± 0.5

### Control Dogs

None of the control dogs had a positive test. All dogs also had a positive reaction to the histamine control (Figure 3A), validating the test (Table 4).

**Table 4:** Number positive AD dogs and control dogs according to the tested allergen.

**Allergen**	**Concentration**	**AD dogs** ** (*n*)**	**Control dogs** ** (*n*)**
Alternaria	3 μg/ml	0	0
Aspergillus	25 μg/ml	0	0
Artemisia vulgaris	50 mg/ml	0	0
Cupressus Arizona	10 mg/ml	2	0
Grass mix (Poa pratensis, Dactilis glomerata, Lolium perenne, Phleum pratense, Festuca pratensis, Helictotrichon pretense)	50 mg/ml	2	0
*Cynodon dactylon*	50 mg/ml	2	0
*Dermatophagoides farinae*	100 mg/ml	4	0
*Dermatophagoides pteronyssinus*	100 mg/ml	4	0
*Blomia tropicalis*	150 mg/ml	5	0
Fire ant	1:100 w/v	2	0
Cat epithelium	10.000 BAU/ml	0	0
Histamine (Positive control)	10 mg/ml	9	9
Dilute saline solution negative control	1:20 w/v	0	0

### Sensitivity and Specificity of the SPT in This Population

For this group of animals, the sensitivity of the prick test was 66%, with a 95% CI of 29.9–92.5% and a specificity of 100% with a 95% CI of 66.3–100%.

### Comparison of the Mean Diameter of Histamine Reaction Between AD Dogs and Controls

The *U*-value is 22.5. The critical value of *U* at *p* < 0.05 was 21. Therefore, the result was not statistically significant (*p* > 0.05), indicating there was no statistical difference in diameters of histamine wheal reactions between atopic dogs and healthy dogs, validating the test.

## Discussion

According to the available literature, this is the first work in which SPT is evaluated in canine AD patients for the diagnosis of the allergens they are sensitized to, where indoors-related allergens were the most common sensitizing agents related to AD in these patients. Traditionally, IDT has been used as the primary screening tool for the implementation of allergen-specific immunotherapy (ASIT) in dogs and cats suffering from AD, contrary to human medicine, where prick test is the first test for the diagnosis of IgE mediated allergic diseases (15). This fact could be owed to the thought that having a dog or cat remains calm and quiet during the test is difficult, and the lack of studies on the use of SPT in dogs providing reference values (15). On the contrary, IDT has become the standard allergy test due to the ease of its performing, once the dog is sedated. The requirement of sedation is one of the disadvantages of IDT compared to SPT, where no sedation is needed, representing an excellent advantage for the patient. *In vitro* measurement of serum IgE specific antibodies has become an essential complementary tool in the diagnosis of type I allergy; however, seldom correlation exists between the results of serum IgE levels and the IDT reaction (14). Because of these differences, none of the above-mentioned methods can be considered a gold standard for the diagnosis of canine AD, and a positive reaction would infer exposure to the allergen, but it is not always associated with clinical symptoms. Currently, these tests are recommended solely to treat clinically compatible atopic dogs with ASIT, according to the results by Hensel et al. (8).

Each patient can have specific IgE (atopy) to different allergens according to the allergenic sources that surround it. Additionally, due to the lack of standardization at the proper concentration to test some allergens, it is possible to find negative tests for a low tested concentration or positive tests due to an irritant effect (8). In our study, the possibility of irritation was ruled out because none of the controls had a positive test. Careful interpretation of the results must be considered along with the clinical history of the dog to contemplate ASIT. A negative result of one trial does not necessarily mean the animal will not be allergic to it in the future (21). Food allergens were not used in this trial because food allergy in dogs is ruled out through a diet trial at the beginning of the diagnostic process in canine atopic dermatitis.

In this study, three dogs with clinical signs compatible with AD did not react to any of the allergens tested. This could be attributed to the fact that the allergens causing the disease were not included in the ones used in this test, or patients had non-atopic dermatitis. Another possibility is that the concentration of the allergen extracts may not have been high enough to induce a positive reaction in these dogs. A 66% sensitivity and a 100% specificity in this group examined warrants further research into prick test as a screening tool for dogs that may be misdiagnosed clinically as having atopic dermatitis. However, the threshold concentrations and comparisons to other tests must be performed in a representative number of dogs to obtain true positive and negative predictive values for the test. *Blomia tropicalis* was the allergen with the highest reactivity among the allergens, seconded by *D. farinae* and *D. pteronyssinus*. These results are similar to those found in the human population of the area, where house dust mites are the primary source of IgE sensitization and allergies (22).

SPT has been the primary tool in humans for the diagnosis of type I allergy showing the best positive predictive value to determine clinical allergy (9). The concordance between *in vitro* specific IgE antibody assays and SPT of 85 and 95% in humans, depending on the allergen being utilized, make it a reliable test with the caveat that SPT provides immediate information vs. *in vitro* test. SPT in dogs with atopic dermatitis is only recently being looked into, but further investigation is needed in order to make significant correlations between the results obtained and the causative offending allergens (23–25). In order to obtain sensitivity and specificity percentages for the test, a comparison between specific serum IgE levels and prick results are necessary. More research is needed in order to validate the sensitivity and specificity of the test obtained in the population studied. Irritant thresholds in a significant number of dogs should be tested in order to have more conclusive results. The observed results support the hypothesis that the coexistence between humans and dogs causes them to have sources of IgE sensitization in common; the frequencies found were similar to previous reports made in the tropics and the study area in humans (26, 27)

A critical aspect of the present study was the use of allergens with a known concentration. There is high variability in the commercially available SPT formulation, particularly of those composed of natural extracts (28). This fact could imply a bias in the results of clinical use of SPT formulations, because of its uncertainty in inducing the response of basophils to the stimuli, and the impossibility to achieve standardized levels of the allergen to be tested for an accurate diagnosis of the test (28). Our results were obtained with well-known concentrations of allergens in a commercial preparation used for routine diagnosis in human SPT (Table 2). Similar differential results on the SPT response according to the source of allergens were found in human patients suffering from atopic dermatitis, which showed differential responses, and authors conclude that the accuracy of SPT relay on the f source of allergen extracts (24). In the study by Carnett and Plant performed in dogs, the authors reported the most appropriate concentration to be used when testing for pollen allergens in dogs. The authors reported a 1/20 dilution for this type of allergens, which is consistent with the concentration used in our study (17).

On the contrary, the concentration we used for testing dust mite-derived allergens of *D. farinae* and *D. pteronyssinus* was higher (100 mg/ml) than the 20 mg/ml report by Carnett and Plant (17). Other authors reported different concentrations of units (w/v vs. ng/ml), which make it impossible to establish comparisons with our results.

In this study we could not evaluate the irritant threshold concentration (ITC) as indicated by Foust-Wheatcraft et al. (18), and other authors, who suggest there is a high variability of ICT of allergens used in SPT depending on the manufacturer, the type of allergens, their source and (18, 20, 29). On the other hand, we used a higher concentration of histamine as a positive control than the optimal concentration reported by Hensel et al. (20), although we do not observe excessive reactions in the positive control in our dogs of study.

Interestingly, several reports on the use of SPT for allergens diagnosis have been performed in healthy dogs, whereas in our study, we used dogs with a long story of clinical symptoms compatible with CAD. We prefer to use this diagnosis with caution because no serum diagnostic test was performed in our patients for measurements of serum IgE levels. Accordingly, in the report by Thom et al. (30), the authors argued in favor of defining quality assurance programs to confirm the reliability of allergen-specific IgE serum measurement in veterinary medicine (30). Curiously, these authors have not deserved the appropriate citation in the literature despite their findings showing the variability of results between laboratories using the same set allergens and the similarity of optical density (OD) results for most of the allergens tested. Accordingly, Lauber et al. (25), questioned the validity IgE in the pathogenesis of CAD in a study that showed no correlations between dust mite extracts and serum IgE levels, and variation of IgE levels depending on breed and castration status of the dog (2, 25). Similarly, Bjelland et al. found a high level of variability in IgE serum levels depending on age, dogs' geographical localization, the season of sampling, and sex, as well as the indoor or outdoor nature of allergens (23).

Finally, in this study, the dogs did not receive sedation, a fact representing one of the advantages of the SPT compared to IDT. After this study, one of the authors has used the SPT regularly for diagnosis purposes in almost 100 dogs, none of them requiring sedation, whatsoever their behavior during the testing time.

The limitations of the study include the small group of animals tested due to the length of the diagnostic process required to rule out any other causes of skin disease, the lack of information because there are no previous studies in the matter regarding to SPT, and the lack of a standardized test in canine atopic dermatitis to utilize as a comparison to SPT. The strength of the study is that the SPT prove to work in a broader population of dogs, it would be a non-invasive (no anesthesia required), and provide the veterinarian with a ready-to-use method of identifying causative allergens in canine atopic dermatitis (CAD), thus accelerating the therapeutic process. Testing in dogs is easy to perform and to interpret. The results in this study using a control group show that it is statistically specific. Should this be proven in a larger-scale study, it would be the right way of ruling-out patients with skin diseases that do not have CAD.

## Conclusions

The lack of information regarding the standardization of allergen concentrations specific to the canine species difficult IDT and SPT interpretation. SPT is a potentially valuable complementary and confirmatory tool in the diagnosis of canine AD. SPT could be a less costly, safer, and more specific test compared to IDT for atopic dogs. House dust mites have consistently been the leading cause of the cutaneous allergic reactions related to AD in dogs.

## Data Availability Statement

The datasets generated for this study are available under request to the corresponding author.

## Ethics Statement

The animal study was reviewed and approved by The University of Antioquia Committee on Animal Subject Experimentation Act of October 6, 2014. Written informed consent was obtained from the owners for the participation of their animals in this study.

## Author Contributions

AC-G and JM-E conceived the study and participate in preparing and reviewing the final version of the manuscript. AC-G recruited, treated, and performed the clinical exams and prick test in dogs of the study. JS participated in preparing and reviewing the final version of the manuscript and corroborates comparisons with human prick test.

### Conflict of Interest

The authors declare that the research was conducted in the absence of any commercial or financial relationships that could be construed as a potential conflict of interest.
